# Basic self-disturbance trajectories in clinical high risk for psychosis: a one-year follow-up study

**DOI:** 10.1007/s00406-021-01349-6

**Published:** 2021-11-16

**Authors:** Tor Gunnar Værnes, Jan Ivar Røssberg, Ingrid Melle, Barnaby Nelson, Kristin Lie Romm, Paul Møller

**Affiliations:** 1 Early Intervention in Psychosis Advisory Unit for South-East Norway, Division of Mental Health and Addiction, Oslo University Hospital, Oslo, Norway; 2 Division of Mental Health and Addiction, Norwegian Centre for Mental Disorders Research, Oslo University Hospital, Oslo, Norway; 3 Psychiatric Research Unit, Division of Mental Health and Addiction, Oslo University Hospital, Oslo, Norway; 4 Institute of Clinical Medicine, Faculty of Medicine, University of Oslo, Oslo, Norway; 5 Orygen, Parkville, VIC Australia; 6 Centre for Youth Mental Health, the University of Melbourne, Parkville, VIC Australia; 7 Institute of Clinical Medicine, Faculty of Medicine, Norwegian Centre for Mental Disorders Research, University of Oslo, Oslo, Norway; 8 Dept. for Mental Health Research and Development, Division of Mental Health and Addiction, Vestre Viken Hospital Trust, Drammen, Norway

**Keywords:** Basic self-disturbance, Anomalous self-experience, Clinical high-risk for psychosis, Schizophrenia spectrum, Functioning, Negative symptoms

## Abstract

Basic self-disturbance (BSD) has been proposed as a driver of symptom development in schizophrenia spectrum disorders (SSDs). In a one-year follow-up of 32 patients (15–30 years) at putative risk for psychosis, we investigated trajectories of BSD levels from baseline to follow-up, and associations between clinical characteristics at baseline and follow-up, including follow-up levels of BSD (assessed with the EASE). Clinical high risk (CHR) for psychosis status and symptom severity were assessed with the SIPS/SOPS scales and also according to the cognitive basic symptoms high-risk criteria (COGDIS). DSM-IV diagnoses, functioning and other clinical characteristics were assessed with standard clinical instruments. Higher severity of negative symptoms and meeting COGDIS criteria at baseline were associated with higher BSD levels at follow-up. All measured at follow-up, higher BSD levels correlated with higher severity of positive, negative, disorganization and general symptoms, and with a lower level of global functioning. We found higher BSD levels at follow-up in subjects with schizotypal personality disorder (SPD) at baseline (*n* = 5) and in SSDs at follow-up (*n* = 12, including nine with SPD). Mean BSD levels decreased significantly from baseline to follow-up, but individual trajectories varied considerably. Increased BSD levels were associated with higher baseline BSD levels, non-remission of positive symptoms and functional decline. Overall, the current study indicates that subgroups in the CHR population with a higher risk of non-remission or deterioration may be identified by supplementing CHR criteria with assessment of BSD and negative symptoms.

**Supplementary Information:**

The online version contains supplementary material available at 10.1007/s00406-021-01349-6.

## Introduction

In a phenomenological model of schizophrenia, first developed by Sass and Parnas, a core feature of this disorder is considered a self-disorder, also termed an ‘ipseity disturbance’ or a ‘basic self-disturbance’ (BSD). The model describes an instability in the basic sense of self, characterized by ‘diminished self-presence’, i.e. disturbances in subjectivity and implicit “ownership” of experience and action, ‘hyperreflexivity’, i.e. an exaggerated self-consciousness involving self-alienation, and ‘disturbed grip or hold’, involving loss of salience, stability and significance of objects in the field of awareness [35, 36, 62, 65, 67]. BSD is assumed to drive symptom development and articulation over the course of the schizophrenia prodrome, and to underlie and connect the seemingly disparate symptoms of all the schizophrenia spectrum disorders (SSDs) [17, 22, 42, 56, 58, 62, 65, 67, 74].

A range of studies have consecutively demonstrated that SSDs and the schizophrenia prodrome are characterized by a panoply of anomalies of self-experience, assumed to reflect BSD [8, 34, 39, 40, 44, 45, 47, 50, 56–58]. These anomalies have been shown to aggregate in SSDs compared to other diagnostic groups and healthy controls, as described in a recent meta-analysis (e.g. SSD vs. bipolar or affective disorders, Hedges *g* = 1.8, CI = 1.4 to 2.2, and SSD vs healthy controls, Hedges *g* = 1.8, CI = 1.5 to 2.0 [57].

To detect, and hopefully prevent, development of psychotic disorders, clinical high-risk (CHR) criteria for psychosis are extensively used in research and clinical settings [21, 69, 72]. The CHR concept is currently based on two different sets of criteria: (1) the ultra-high risk (UHR) criteria, and (2) the basic symptoms high-risk criteria [19, 72]. Several studies have demonstrated that BSD phenomena are common in CHR samples [13, 14, 37, 54, 77], although less frequent than in SSDs [57]. However, prospective studies of BSD in CHR samples are sparse. One study found that a higher level of BSD was associated with transition to psychosis in an UHR sample. Being diagnosed with SSDs (including both psychotic SSDs and schizotypal or schizoid personality disorder) was also associated with higher BSD levels [37]. In a previous communication from the current research project, we found that non-remission of attenuated psychotic symptoms and functional deficits was associated with higher baseline levels of BSD [80]. It could also be noted that in a seven-year follow-up study of non-psychotic help-seeking adolescents (i.e. not restricted to CHR), future SSD diagnoses were significantly predicted by BSD levels [24]. 

To our knowledge, no studies have prospectively investigated the persistence of BSD phenomena in CHR, and how BSD trajectories and BSD levels at follow-up may be related to symptoms, other clinical characteristics and functioning at baseline and follow-up. This is of importance because it may help us identify CHR subjects at the highest risk of adverse clinical and functional outcomes, and to derive a more nuanced picture of the stability of BSD in CHR.

In this exploratory study, our aims were to address the following questions in a one-year follow-up of a CHR sample:

1. To what extent are clinical characteristics and functioning at baseline associated with the severity of BSD at one-year follow-up?
2. To what extent is the severity of BSD at one-year follow-up associated with clinical characteristics and functioning at follow-up?
3. How stable is BSD from baseline to one-year follow-up?
4. Are different BSD trajectories associated with differences in clinical and functional characteristics at baseline, and with changes in these characteristics from baseline to follow-up?

## Methods

### Setting and participants

The present study was a one-year follow-up of patients from child/adolescent and adult outpatient units in Oslo and adjacent catchment areas (Oslo University Hospital, Diakonhjemmet Hospital, Vestre Viken Hospital Trust and Akershus University Hospital). Patients were referred to the study if they were clinically suspected by their treating clinicians to be at increased risk of psychosis, and were consecutively recruited and assessed at baseline from June 2012 to December 2015. All participants gave written informed consent. For those below 18 years, parents consented as well. The study was part of the Norwegian Thematically Organized Psychosis (TOP) study, and was approved by the Regional Committee for Medical Research Ethics in Norway (permission number 2011/1070 D).

Inclusion criteria were age between 15 and 30 years, and meeting CHR criteria for one or more of the following UHR syndromes: the Attenuated Positive Symptom Syndrome (APSS), the Brief Intermittent Psychotic Symptoms (BIPS) syndrome or the Genetic Risk and Deterioration (GRD) syndrome, as outlined in the Structured Interview for Prodromal Syndromes (SIPS) [29] (see Table 1). An APSS syndrome in this SIPS version does not require social/occupational dysfunction, as in the CAARMS attenuated psychosis group [20], or distress/disability, as in the DSM-5 APS syndrome [1, 61].

**Table 1:** UHR/COPS^a^, non-progressive symptoms criteria, and COGDIS^b^ criteria

Prodromal syndromes	Criteria of Prodromal Syndromes (COPS)
*Attenuated Positive Symptom syndrome (APSS)*^c^ Scale of Prodromal Symptoms (SOPS), positive subscale, include: unusual thought content/delusional ideas, suspiciousness/persecutory ideas, grandiosity, perceptual abnormalities/hallucinations and disorganized communication	One or more of the 5 SOPS positive items scoring in the prodromal range (rating of 3–5) AND Symptoms beginning within the past year or currently rate at least one scale point higher than it would if rated 12 months ago AND Symptoms occurring at least once per week for last month
*Brief Intermittent Psychotic Symptom (BIPS) syndrome*	One or more of the 5 SOPS positive items in the psychotic range (rating of 6) that do not meet Presence of Psychotic Syndrome (POPS) criteria in the SIPS AND Symptoms beginning in the past 3 months AND Symptoms occurring currently at least several minutes per day at least once per month
*Genetic Risk and Deterioration (GRD) syndrome*	First degree relative with history of any psychotic disorder OR Criteria for schizotypal personality disorder met in patient AND GAF drop of at least 30% over the last month vs 1 year ago
**Non-progressive symptoms group**	**Criteria for the non-progressive symptoms group**
	One or more of the 5 SOPS positive items scoring in the prodromal range (rating of 3–5) AND Symptoms occurring at least once per week for last month
**COGDIS items**	**COGDIS criteria**
Inability to divide attention, thought interference, thought pressure, thought blockages, disturbance of receptive speech, disturbance of expressive speech, unstable ideas of reference, disturbances of abstract thinking, captivation of attention by details of the visual field	Presence of ≥ 2 of the 9 basic symptoms with a SPI-A score of ≥ 3 within the last 3 months

^a^Descriptions are from the SIPS [30], Norwegian version 5.0 (Jan. 2012)

^b^The listed COGDIS items and criteria are obtained from: Schultze-Lutter F, Addington J, Ruhrmann S, Klosterkötter J [70] Schizophrenia Proneness Instrument – Adult version (SPI-A). Giovanni Fiori Editore, Roma

In addition, we did not exclude patients with longstanding, non-progressive attenuated psychotic symptoms. They met criteria for an APSS syndrome [29], except the recent onset/progression criteria. We termed these subjects the ‘non-progressive symptoms group’. They would possibly have met the criteria for an APSS syndrome with the ‘current status specifier’ ‘persistence’ in the current version of the SIPS (version 5.6) [81]. Subjects with persistent risk symptoms may be at risk of a range of adverse clinical and functional outcomes, although the risk for conversion to psychosis is lower than in CHR subjects with progressive symptoms [3, 18, 68, 81]. All subjects were also assessed with respect to cognitive basic symptoms high-risk criteria (COGDIS) during the following baseline assessments. See Table 1 for detailed descriptions of the UHR, COGDIS and non-progressive symptoms group criteria [70].

We excluded subjects who met one or more of the following criteria: current or past psychotic disorder (DSM-IV Axis 1 criteria), being treated with antipsychotics currently or for ≥ 4 weeks lifetime (dose equivalent to ≥ 5 mg Olanzapine per day), clearly drug-induced CHR symptoms, neurological disorders or severe medical conditions, intellectual disability (IQ < 70), and incapacity to speak/comprehend Norwegian.

The original baseline sample comprised 38 participants, including seven in the non-progressive symptoms group. Six subjects (5 CHR, 1 non-progressive) did not take part in the assessments at follow-up, i.e. a drop-out rate of 15.8%. Hence, 32 subjects took part in the current follow-up study, including six in the non-progressive symptoms group. There were no significant differences in baseline demographic or clinical characteristics between these 32 and the six drop-outs (supplementary material S1. The original baseline sample is also described in a previous study [77]).

### Measures and procedure

#### Baseline assessments

Baseline assessments included socio-demographic data and the Structured Interview for Prodromal Syndromes/Scale of Prodromal Symptoms (SIPS/SOPS) [29, 30], Norwegian version 5.0, Jan 2012. The SIPS/SOPS was used for assessing UHR criteria and non-progressive symptoms criteria, and the last month severity of positive, negative, disorganization and general symptoms (ranging each symptom on the SOPS from 0 = absent to 6 = psychotic/extreme) [31]. Inter-rater reliability regarding SOPS scores and prodromal/psychosis-risk syndrome diagnostic agreement have been found to be excellent in early studies and in a more recent review [29, 30, 82]. Studies are sparse, but also find the predictive and construct validity of the SIPS/SOPS to be satisfactory [82]. The non-progressive symptoms criteria were not tested for validity and reliability. Considering the overlap with criteria for the CHR “persistence” syndrome, it could be noted that this new CHR classification system has shown promising validity [81].

BSD phenomena were assessed with the Examination of Anomalous Self-Experiences (EASE) (lifetime experiences). The EASE comprises 57 main items organized in five domains: (1) Cognition and stream of consciousness, (2) Self-Awareness and presence, (3) Bodily experiences, (4) Demarcation/Transitivism, and (5) Existential reorientation (supplementary material S2) [49]. All EASE items were scored on a 0–4 severity Likert scale, but following other similar studies [24, 39, 54] we subsequently converted these scores into dichotomous 0–1 scores, indicating that the symptom had been absent or questionably present (0), or definitively present (1). The EASE has been found to have good to excellent internal consistency and inter-rater reliability [33, 37, 41, 55]. SIPS and EASE interviews were videotaped at baseline and follow-up. Based on retrospective inspection of the baseline EASE interviews, we did an additional baseline scoring of all the EASE items (0–1 scores), reflecting present or last year experiences. COGDIS criteria were assessed according to descriptions in the Schizophrenia Proneness Instrument – Adult version (SPI-A) [70], using all available information including the EASE and SIPS interviews. There is a considerable overlap between the descriptions of the COGDIS symptoms in the SPI-A and certain EASE items [49, 70]. The SPI-A has demonstrated good inter-rater reliability [71], and the predictive validity of the COGDIS criteria is comparable to the UHR criteria [60, 72].

Clinical DSM-IV Axis I diagnoses were allocated after an assessment with a full version of the SCID-I [16]. A checklist included in the SIPS for the DSM-IV criteria for Schizotypal Personality Disorder (SPD) was used for assessment of this disorder. We categorized SPD as schizophrenia spectrum disorder, in line with DSM-5 and the understanding of SPD among experts in the field [1, 15, 48, 73]. Present (last week) global functioning was assessed with a split version of the Global Assessment of Functioning scale (S-GAF). S-GAF is divided into a symptom score and a functioning score, ranging in severity from 0 (extreme dysfunction) to 100 (superior function) [51]. Only the functioning scores (GAF-F) are reported here. Childhood (0–11 years) and early adolescent (12–15 years) functioning were assessed with the Premorbid Adjustment Scale (PAS) [9], and adverse childhood experiences with the self-report inventory Childhood Trauma Questionnaire – short form (CTQ-SF) [6]. CTQ-SF include 28 items and categorize experiences in five domains: physical abuse, sexual abuse, emotional abuse, emotional neglect and physical neglect [5].

#### Follow-up assessments

At follow-up, we did a reassessment with the SIPS/SOPS (based on last month), the EASE (covering last year, since baseline) and GAF-F (based on last week). In line with a recent recommendation from clinical and research experts in the field [52], we used a combined symptomatic and functional measure of remission. This was defined as a score of ≤ 2 on all SOPS positive symptom items, in combination with a score of ≥ 70 points or ≥ 10 points improvement on GAF-F (corresponding, but not identical, to the measure suggested by Polari et al. [52]). In the case of transition to psychosis between baseline and follow-up (reported from treating clinicians), this was evaluated according to the criteria for a psychotic syndrome in the SIPS [29, 30], followed by a differential diagnostic assessment with the SCID-I, module 1, A-E chapters [16]. Non-transitioning subjects were not reassessed with the SCID-I, but all participants were reassessed at follow-up with the SPD checklist in the SIPS.

Clinical interviews at baseline and follow-up were performed by TGV. He had participated in the TOP study SCID-I reliability and training program, and had been trained in the use of the SIPS and EASE by Norwegian experts in the field, including supervision in the use of EASE by PM, one of the authors and certified instructors of the EASE. Inter-rater reliability was tested on the SIPS and EASE, revealing excellent reliability for the SIPS and moderate reliability for the EASE (for details, see [77]). DSM-IV diagnoses, CHR status and EASE scores were regularly discussed with PM and JIR, both experienced researchers and psychiatrists.

### Statistical analysis

All statistical analyses were performed with SPSS version 25.0. Non-parametric tests were used, and if not otherwise specified, the significance threshold was set at 0.05. The severity level of BSD was determined by summing up the dichotomous 0–1 scores on all the 57 main EASE items, giving an EASE total score. Likewise, the severity level of positive, negative, disorganization and general symptoms was determined by summing up the scores on the SOPS subscales. All tests of normality of the distribution of scores were conducted with the Kolmogorov–Smirnov statistic, and we inspected skewness and kurtosis values. Group comparisons of categorical variables were conducted with chi-square statistics.

The EASE total scores at follow-up were positively skewed, clustering at the low values. Correlations between EASE total at follow-up and continuous variables at baseline (first research question) and follow-up (second research question) were tested with Spearman’s rho (two-tailed). These analyses were Bonferroni-adjusted for multiple comparisons (alpha level *p* < 0.006 (0.05/9 variables) in the first analysis, and *p* < 0.01 (0.05/5 variables) in the second analysis. In the first analysis, we included the CTQ subscale Emotional neglect, but not the other CTQ subscales, given a stronger association (*p* < 0.05) with EASE total at baseline [77]. Analyses of whether differences in EASE total at follow-up were associated with categorical variables at baseline or follow-up were conducted with the Mann–Whitney U Test.

To answer the third and fourth research question, we used the Wilcoxon signed rank test to analyze differences in EASE total between baseline and follow-up. Baseline EASE total scores based on current/last year experiences were included in these analysis (thus comparing the presence of anomalous self-experiences from one year to the next year). One outlier with the strongest increase in EASE scores was included in the analyses, as the inclusion of this outlier did not significantly affect the results.

By inspection of the individual EASE trajectories, we did an ad hoc categorization into three groups: 1) subjects with an *increase* (≥ 1 points) in EASE total, 2) subjects with *0–3 points decline* in EASE total and 3) subjects with > *3 points decline* in EASE total. We chose this approach over statistical clustering approaches due to the small sample size. Four SOPS subscale change variables and a GAF-F change variable were computed (follow-up minus baseline scores). Differences between the three groups in the scores on the baseline variables and the scores on the SOPS change and GAF-F change variables were analyzed with the Kruskal–Wallis test.

## Results

### Demographics and clinical characteristics at baseline and one-year follow-up

Twenty-six participants were meeting formal CHR criteria, and six were assessed as ‘non-progressive’, at baseline. A majority (*n* = 24, 92%) met criteria for an APSS syndrome, either alone or in combination with COGDIS criteria or a GRD syndrome (only one). In Table 2, demographic and clinical characteristics of the sample (*n* = 32) are shown. The six participants in the non-progressive symptoms group did not differ significantly from the CHR group in clinical or demographic characteristics, except for being approximately five years older and having approximately one more year of education (supplementary material S3). Medication at baseline had no association to clinical variables at baseline.

**Table 2:** Demographics and clinical characteristics at baseline and one-year follow-up

Characteristics	Baseline	Follow-up
Participants, *n*	32	32
Male, *n* (%)	21 (65.6)	
Age, mean (*SD*)	19.9 (3.8)	21.1 (4.0)
Born in Norway, *n* (%)	29 (90.6)	
Employed or studying, *n* (%)	17 (53.1)	
Years of education, mean (*SD*)	11.7 (1.8)	
CHR positive, *n* (%), including:	26 (81.3)	9 (28.1)^a^
APS only	14 (43.8)	2 (6.2)
APS + COGDIS	9 (28.1)	1 (3.1)
APS + GRD + COGDIS	1 (3.1)	1 (3.1)
COGDIS only	2 (6.2)	5 (15.6)
Non-progressive SOPS pos, *n* (%)	6 (18.8)	4 (66.7)
Transition to psychosis, *n* (%)		4 (12.5)
Symptomatic and functional remission, *n* (%)^b^		11 (34.4)
SOPS (number of items)		
Positive (5), mean (*SD*)	10.41 (3.45)	6.56 (5.58)
Negative (6), mean (*SD*)	12.50 (7.02)	9.94 (7.39)
Disorganization (4), mean (*SD*)	6.91 (3.36)	5.13 (4.32)
General (4), mean (*SD*)	7.59 (3.31)	4.97 (3.49)
EASE total, mean (*SD*)/median		
Baseline: lifetime	15.31 (8.01) /13.50	
Baseline: last year	13.78 (8.06) /12.00	11.09 (10.03)/8.50
GAF-F, mean (*SD*)	56.31 (10.83)	59.80 (15.72)
Diagnoses		
Mood disorders, *n* (%)	13 (40.6)	
Anxiety disorders, *n* (%)	8 (25.0)	
Other Axis 1 disorders, *n* (%)	4 (12.5)	
Schizotypal pers. dis., *n* (%)	5 (15.6)	9 (28.1)
Schizophrenia, *n* (%)		2 (6.2)
Schizophreniform disorder, *n* (%)		1 (3.1)
Psychosis NOS, *n* (%)		1 (3.1)
No DSM-IV diagnosis, *n* (%)	2 (6.3)	
Medication, prescribed^c^, *n* (%)		
Antipsychotics	7 (21.9)	8^d^ (25.0)
Antidepressants	6 (18.8)	10 (31.3)
Anxiolytic	2 (6.3)	0
Anticonvulsants	1 (3.1)	1 (3.1)
Psychostimulants	1 (3.1)	1 (3.1)
Hospitalization between baseline and follow-up, *n* (%)		3 (9.4)
Discontinuation of treatment before follow-up,* n* (%)		8 (25)

^a^Meeting full CHR criteria, e.g. worsening of attenuated positive symptoms last year

^b^≤ 2 on all SOPS positive symptom items, in combination with a score of ≥ 70 points or ≥ 10 points improvement on GAF-F. Two of the 11 remitted subjects were from the non-progressive symptoms group

^c^Data in the follow-up column represents prescribed medication *between* baseline and follow-up

^d^5 of the 8 had “Defined Daily Dose” below the recommended for antipsychotic treatment

The mean follow-up time was 13 months (*Sd* = 1.7). The participants received treatment as usual at their local health services between baseline and follow-up, including standard medication, psychotherapy and psychosocial interventions (e.g. family support and work/school adjustments). Outcomes at follow-up were not significantly affected by these treatment variables, or by hospitalizations or discontinuation of treatment. Investigations of relationships between demographic characteristics and clinical and functional outcomes at baseline or follow-up did not reveal any significant associations.

Among the four participants who transitioned to psychosis, three were assigned a DSM-IV SSD diagnosis (2 schizophrenia, 1 schizophreniform disorder). The fourth was diagnosed with DSM-IV Psychosis NOS. Nine were diagnosed with SPD at follow-up (increased from five at baseline). We categorized these nine as schizophrenia spectrum subjects, along with the three with schizophrenia and schizophreniform disorder (*n* = 12, i.e. 37.5% of the sample).

### Clinical characteristics at baseline were associated with EASE total at follow-up

Correlations between baseline variables and EASE total at follow-up are shown in Table 3. The scores on the SOPS negative, SOPS disorganization and PAS Early Adolescence subscales correlated with EASE total at a significance level of *p* < 0.05, but after Bonferroni-correction (*p* < 0.006), only the association with SOPS negative was statistically significant, with a large effect size (*r* = 0.58).

**Table 3:** Correlations between clinical and demographic characteristics at baseline and EASE total at one-year follow-up (*n* = 32)

Baseline variables ** → **	SOPS Pos	SOPS Neg	SOPS Disorg	SOPS Gen	GAF-F	CTQ total	CTQ Emot. Negl	PAS Childhood	PAS Early adol
EASE total at 1 year	0.17	0.58**	0.46*	0.20	− 0.30	0.11	0.26	0.22	0.38*

*p < .05, **p < .006 (Bonferroni-adjusted), Spearmans rho, two-tailed

Subjects meeting COGDIS criteria (*n* = 12) had significantly higher follow-up EASE total scores (*Md* = 18) than the other participants (*n* = 20, *Md* = 4.5), *U* = 59.5, *p* = 0.02, with a medium effect size (*r* = 0.42). This difference remained significant when EASE items clearly overlapping with COGDIS items (EASE items 1.1, 1.3, 1.4, 1.12, 1.17 and 5.1) were removed from the EASE total score, *U* = 64, *p* = 0.03. Baseline SPD subjects also had significantly higher EASE total scores at follow-up (*n* = 5, *Md* = 21) than the other subjects (*n* = 27, *Md* = 6), *U* = 28.5, *p* = 0.04, *r* = 0.36.

### Clinical characteristics and functioning at follow-up was associated with EASE total at follow-up

All SOPS subscales and GAF-F at follow-up were significantly associated with EASE total at follow-up (Table 4), with large effect sizes (*r* > 0.60) for all these correlations.

**Table 4:** Correlations between EASE total and clinical characteristics at follow-up (*n* = 32)

Measures at f-u →	SOPS pos	SOPS neg	SOPS disorg	SOPS gen	GAF-F
EASE total at f-u	0.75*	0.76*	0.75*	0.64*	− 0.79*

*p < .01 (Bonferroni-adjusted), Spearmans rho, two-tailed

SSD subjects (*n* = 12, at follow-up) had significantly higher EASE total scores at follow-up (*Md* = 16.5) than subjects with no SSD (*n* = 20, *Md* = 4.5), *U* = 194.5, *p* = 0.003, *r* = 0.51 (highest in the three SSDs with psychotic disorders: *Md* = 28, SPD subjects: *Md* = 12). These SSD subjects also scored significantly higher on all the SOPS subscales at follow-up (SOPS positive and SOPS disorganization: *p* < 0.001, SOPS negative: *p* = 0.004), except for SOPS general (*p* = 0.08), and at a significantly lower level of GAF-F (*p* = 0.003). Among non-remitted subjects (*n* = 21), eleven were diagnosed with SSDs (9 of 11 with SPD) at follow-up. The non-remitted subjects (*n* = 21) had significantly higher EASE total scores at follow-up (*Md* = 14.5) than the fully remitted subjects (*n* = 11, *Md* = 1), *U* = 16.5, *p* < 0.001.

### EASE level trajectories, and their associations with clinical and other characteristics from baseline to follow-up

**Fig. 1:**
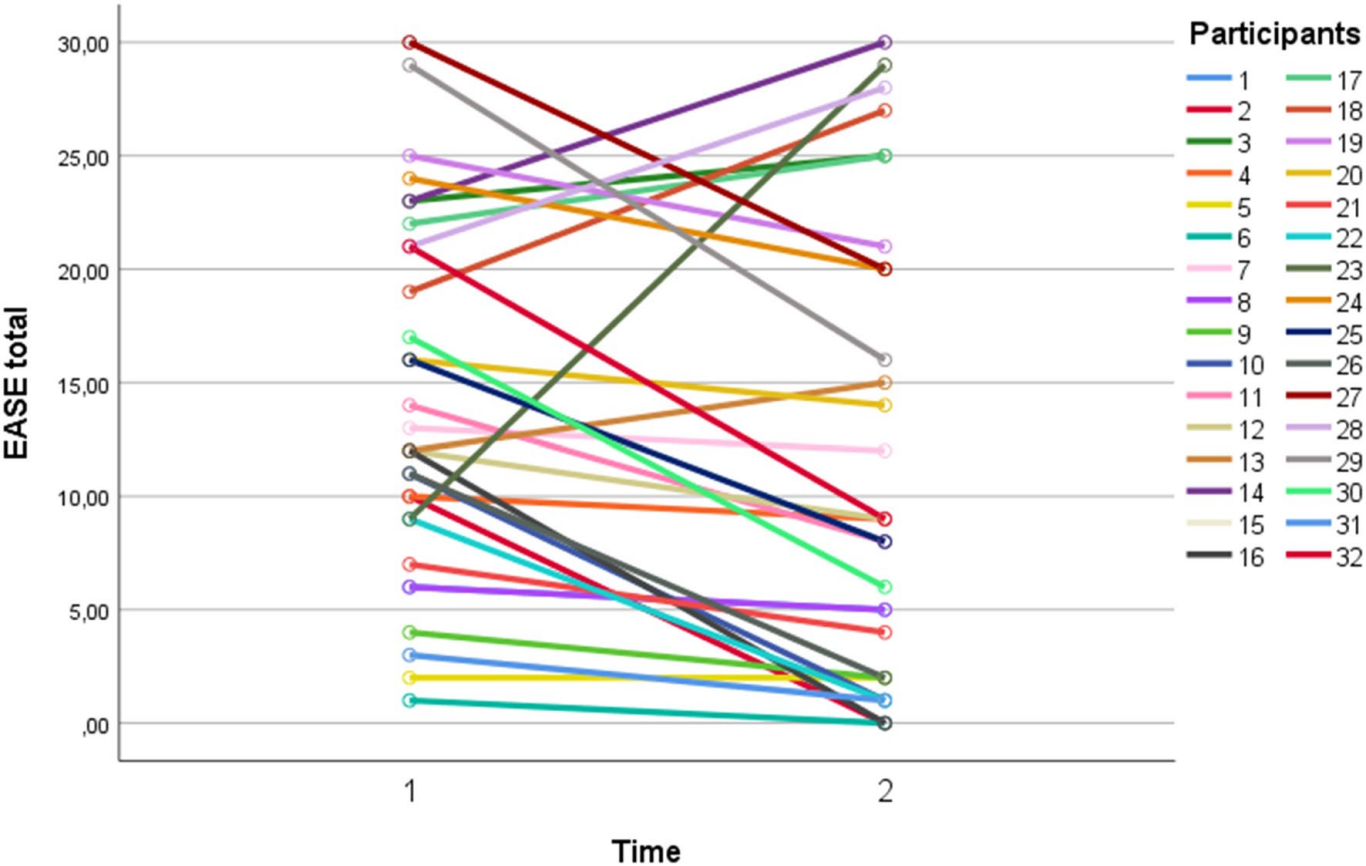
Individual trajectories in mean EASE total scores from baseline to follow-up

Individual trajectories of EASE scores from baseline to follow-up are illustrated in Fig. 1. Median EASE total in the full sample decreased from 12 at baseline to 8.5 at follow-up. This was a significant decline (*z* = -2.47, *p* = 0.01), with a moderate effect size (*r* = 0.31). There was one outlier with a very strong increase in EASE total (see Fig. 1). The significant decline in EASE total scores from baseline to follow-up was found in females (*z* = -2.94, *p* = 0.003), but not in males (*z* = -1.81, *p* = 0.07). Males scored nominally higher on EASE total at follow-up (*Md* = 12, *n* = 21) than females (*Md* = 4, *n* = 11), but this difference was not significant (*U* = 163.5, *p* = 0.06).

**Fig. 2:**
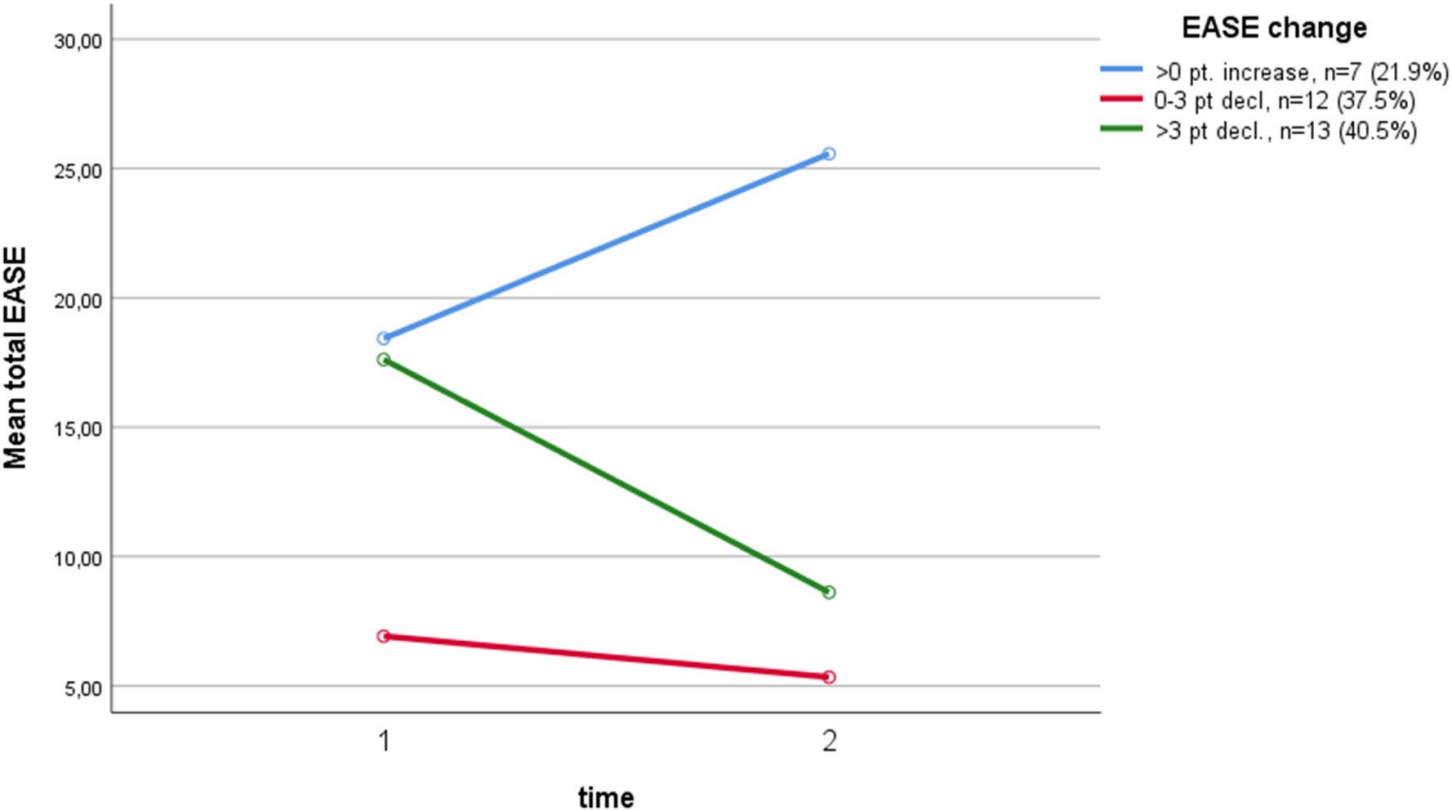
Mean changes in EASE total scores for the three EASE trajectories groups

Breaking the total sample down into three groups, seven subjects (21.9%) had an ≥ 1 point increase in EASE total from baseline (*Md* = 21) to follow-up (*Md* = 27), twelve subjects (37.5%) had a 0–3 points decline (baseline: *Md* = 6, follow-up: *Md* = 4.5), and thirteen subjects (40.6%) had a > 3 points decline (baseline: *Md* = 16, follow-up: *Md* = 8). The mean changes in EASE total scores for the three groups are illustrated in Fig. 2. Patients diagnosed with SSDs at follow-up (*n* = 12) did not have a significant decline in EASE total (baseline: *Md* = 18.5, follow-up: *Md* = 16.5), *z* = -0.45, *p* = 0.96. However, SSD subjects were found in all three groups: five increased, four declined 0–3 points, and three declined > 3 points.

Analyses revealed a statistically significant difference in baseline EASE levels between the three groups, χ^2^ (2, *n* = 32) = 14.06, *p* = 0.001. Post hoc comparison tests (Mann Whitney U) revealed that the median EASE total score for the ‘EASE 0–3 points decline group’ (*Md* = 6) was significantly lower than the median score in both the ‘EASE increase group’ (*Md* = 21, *p* = 0.007) and the ‘EASE > 3 points decline group’ (*Md* = 16, *p* = 0.003). Further analyses showed no other significant differences in baseline characteristics between the three groups.

Analyses of differences between the three trajectory groups revealed significant differences in SOPS positive change (χ^2^ (2, *n* = 32) = 11.25, *p* = 0.004) and in GAF-F change (χ^2^ (2, *n* = 32) =  = 9.11, *p* = 0.01), but not in the other three SOPS change variables. Mean change scores for the three groups are illustrated in Table 5. As can be seen, there was a nominal *increase* in positive symptoms and a decreased functioning in the EASE increase group. This contrasted significantly with the *decrease* in positive symptoms and the increased functioning at follow-up in the two other groups.

**Table 5:** Changes in SOPS symptoms and GAF-F in three EASE change groups

	SOPS positive change *M (SD)*	SOPS negative change *M (SD)*	SOPS disorg change *M (SD)*	SOPS general change *M (SD)*	GAF-F change *M (SD)*
EASE increase (≥ 1 pt)	2.00 (5.69)	− 0.86 (6.28)	0.71 (4.46)	− 1.29 (4.31)	− 8.43 (8.60)
EASE 0–3 pt decline	− 4.08 (4.91)	− 1.33 (4.21)	− 1.92 (2.88)	− 2.08 (4.38)	7.83 (10.04)
EASE > 3 pt decline	− 6.77 (3.30)	− 4.62 (3.62)	− 3.00 (2.16)	− 3.85 (3.74)	5.77 (10.58)
Exact *p* value^a^	0.004*	0.085	0.052	0.273	0.011*

^a^Kruskal Wallis test, *p < .05

## Discussion

### Baseline characteristics and EASE total at follow-up

The strong, positive correlation between baseline negative symptoms and BSD levels at one-year follow-up was in line with the strong association between negative symptoms and BSD levels at baseline found in a previous study of this sample (also including the six drop-outs) [77]. This may imply that a higher severity of negative symptoms in CHR may be associated with a higher probability of sustained or recurring high BSD levels as future outcomes. However, this of course does not necessarily mean that negative symptoms *cause* BSD, or vice versa. Phenomenologically oriented theories suggest that negative symptoms (along with other clinical manifestations) are meaningful, intimately interconnected aspects of an underlying psychopathological”Gestalt”, characterized by disturbances in the structure of subjectivity, i.e. BSD [42, 64, 67]. Basic symptoms may also constitute such aspects [43–45], thus possibly explaining that subjects meeting COGDIS criteria at baseline had higher levels of BSD at follow-up than the other participants (also when removing EASE items from the analyses clearly overlapping with the COGDIS items). The significantly higher BSD levels at follow-up, as well as at baseline [77], in subjects assessed with SPD at baseline, were in line with other studies demonstrating that SPD and ICD-10 schizotypal disorder are associated with BSD levels markedly higher than in conditions outside of the schizophrenia spectrum [22, 40, 55, 57].

The results indicated better outcomes in females than males with respect to future BSD levels (a significant decline). Studies have found more severe negative symptoms and poorer social functioning in CHR males [2, 59], which are characteristics associated with poorer clinical and functional prognosis in several studies, e.g. [4, 10, 25, 68]. Considering that BSD levels and negative symptoms were strongly associated in this study, the better outcome in females seems not surprising. However, the severity of negative symptoms was only nominally higher in males at both time points. Gender differences are underexplored as a research topic in CHR studies, and have been found to be rather small [28]. Hence, the differences found in the current and other studies should be investigated in larger samples.

We can only speculate about the lack of a significant effect of medication and other aspects of treatment on EASE total and other clinical variables at baseline and follow-up. The small sample size may have diminished the probability of finding such effects. The effect of medication on BSD is another underexplored field, and for the majority of subjects prescribed antipsychotics, daily doses were considerably below what is considered having an antipsychotic effect.

### EASE total at follow-up vs. other characteristics at follow-up

The strong associations at follow-up between high BSD levels, lower level of global functioning and higher severity of symptoms on all SOPS subscales, point to a consolidation of a psychopathological Gestalt with BSD as a core feature, accruing as time has passed. The more severe clinical pattern found in SSD subjects fits well with the BSD/ipseity disturbance model [36, 42, 48, 65, 67]. An alternative hypothesis is that BSD may be a marker of elevated (severe) levels of a “general psychopathology” (*p*) factor crossing symptomatic domains and diagnostic boundaries [11], increasing the risk of the psychopathological expressions typically found in the SSDs.

Our results contrast to some extent with the findings in a 5-year follow-up study, investigating associations between BSD levels, positive and negative symptoms, and functioning in schizophrenia spectrum patients [39]. In this study, only positive symptoms at follow-up correlated with BSD levels at follow-up. In addition, significant correlations were found between baseline BSD and global symptom levels at baseline and follow-up [39]. It is likely that the difference between these two studies is due to stronger diagnostic homogeneity and higher severity of the sample in the 5-year follow-up study, in comparison with the heterogeneous CHR sample in the current study.

A possible explanation for the weaker correlations between baseline SOPS subscale and GAF-F variables and BSD levels at follow-up, compared to the correlations only including follow-up equivalents of these variables, could be that the symptoms measured by SOPS and GAF are affected by many other factors than BSD in early CHR conditions. Attenuated psychotic symptoms are not uncommon in youth with mental health concerns and functional decline [12, 76, 79], and not even in the general population [23, 26]. These attenuated symptoms may constitute quite non-specific reactions to stressful conditions, rather than be driven by BSD [32]. Hence, they may also be of a transient or fluctuating nature in many CHR subjects. In addition, weaker correlations at baseline between BSD and positive symptoms may reflect a more restricted range of positive symptoms at baseline, due to the inclusion criteria. Dysfunction in CHR may also vary and improve as time unfolds [68], though it may also have a non-remitting or even deteriorating course in these conditions [38, 68].

### Changes in EASE total and associations with other characteristics

Median BSD levels decreased in the total sample, but individual BSD trajectories varied considerably. This might indicate that BSD is not unconditionally trait-like and stable in CHR conditions. According to a recent suggested revision of the self-disorder model [7, 62, 66], some BSD phenomena may have a ‘secondary’, reactive, state-like quality, due to the interaction between adverse environmental circumstances and individual vulnerabilities. These are assumed to occur in SSDs, but also in dissociative and anxiety conditions [27, 63, 78]. Possibly, they are also frequent in CHR conditions. Other BSD phenomena may be more ‘primary’, ‘automatic’, stable features, possibly reflecting early neurodevelopmental disturbances [53]. These may be more specific to SSDs and prodromal schizophrenia. Individual differences in the predominance of primary versus secondary BSD phenomena could possibly manifest in different BSD trajectories.

As a group, subjects with SSD diagnoses at follow-up did not show a significant decline in BSD levels, but some had and increase while others had a small or a marked decline. The EASE increase group (5 of 7 with SSDs) was characterized by a more severe clinical pattern, including higher baseline levels of BSD, and symptomatic and functional non-remission at follow-up. Svendsen et al. [75] also found increasing, stable and decreasing BSD levels in patients with schizophrenia. As suggested by these authors, BSD levels may be more influenced by individual characteristics, including response to treatment, than previously thought [8, 46]. In the current study, subjects with a 0–3 points decline in EASE total had significantly lower BSD levels at baseline than the other participants. Given the low levels at baseline, which implies a good prognostic sign, it is not surprising that these levels were still low and stable at follow-up.

The revised BSD model remains to be properly tested. This would require prospective studies in larger samples than the current study, investigating the presence and stability of BSD in patients from different diagnostic groups, and addressing both intra-individual patterns and inter-individual differences. It should also be noted that changes in EASE scores may not necessarily reflect more or less anomalous self-experiences, but may also be due to variations in the “availability” (mental awareness) of experiences for the person, and the ability to communicate them [34, 49].

## Limitations

The firmness of the conclusions is restricted due to the small sample size, which also included the ‘non-progressive symptoms group’. We partly controlled for this limitation by doing all analyses with and without the ‘non-progressive’ subjects, and this did not affect the results. Analyses comparing the small subgroups in the sample may have increased the risk of type 1 and type II errors. Including a larger control group of help-seeking non-CHR subjects with no positive symptom inclusion criterion, would have been appropriate to avoid the problem of the restricted range of positive symptoms at baseline. This would also have increased the possibility of doing comparative analyses, and thus the generalizability of the results. The ad hoc approach to the categorization of BSD trajectories in three groups is another limitation. Finally, the rater doing the follow-up assessments should have been blind to the baseline findings. On the other hand, this is to our knowledge the first CHR study investigating with the full EASE scale at two time points. In light of the small sample, findings are primarily of interest to generate hypotheses well worth investigating in larger samples.

## Conclusions

This study found that CHR subjects characterized by more severe negative symptoms, cognitive disturbances and higher BSD levels at baseline were particularly vulnerable for a consolidation of a comprehensive psychopathological Gestalt as time passed, with BSD as a core feature. In line with the BSD model, this consolidation was more common in subjects with SSDs (9 of 12 with SPD) at follow-up. The general decrease in BSD levels, together with the individual variations in BSD trajectories, indicated that BSD phenomena in CHR conditions may vary with respect to having a state-like or a trait-like character, in line with the suggested revision of the self-disorder model [62]. Increasing BSD levels may constitute a marker of a non-remitting or even progressively worsening symptomatic and functional course. Taken together, the results demonstrated that longitudinal investigations of BSD are helpful in identifying CHR subjects at particularly high risk for adverse symptomatic and functional outcomes, even in non-converting to psychosis cases. If replicated in prospective CHR studies with larger samples, these findings may contribute considerably to the clinical identification of such particularly vulnerable CHR subjects.

## Supplementary Information

Below is the link to the electronic supplementary material.

Supplementary file1 (DOCX 25 KB)
