# Supplementary material for: Basic self-disturbance trajectories in clinical high risk for psychosis: a one-year follow-up study
Source: Eur Arch Psychiatry Clin Neurosci. 2021 Nov 16;272(6):1007–19. doi: 10.1007/s00406-021-01349-6 (PMC9388413; doi:10.1007/s00406-021-01349-6)
Supplement: Supplementary file 1 — Supplementary file1 (DOCX 25 KB) [file 406_2021_1349_MOESM1_ESM.docx]

**S1 Comparison of demographics and clinical characteristics at baseline in the follow-up study sample and in the drop-outs**

| Demographic and characteristics at baseline | Sample in follow-up study  *N* = 32 | Drop-outs  *N* = 6 | *P-*value |
| --- | --- | --- | --- |
| Male, *n* (%)  Age, mean (*SD*)  Years of education, mean (*SD*  Employed or studying, *n* (%)  Born in Norway, *n* (%)  CHR positive, *n* (%)  EASE total (lifetime), mean (*SD*)  EASE total (last year), mean (*SD*)  SOPS positive, mean (*SD*)  SOPS negative, mean (*SD*)  SOPS disorganization, mean (*SD*)  SOPS general, mean (*SD*)  GAF-F, mean (*SD*) | 21 (65.6)  19.9 (3.83)  11.3 (1.03)  17 (53.1)  29 (90.6)  26 (81.3)  15.31 (8.13)  13.78 (8.06)  10.41 (3.45)  12.50 (7.02)  6.91 (3.36)  7.59 (3.31)  56.31 (10.83) | 3 (50.0)  19.2 (2.64)  11.7 (1.81)  3 (50.0)  5 (83.3)  5 (83.3)  16.17 (10.03)  14.83 (10.42)  8.67 (3.72)  15.33 (6.88)  7.00 (2.97)  10.17 (3.82)  52.83 (12.67) | NSᵃ  .86ᵇ  .74ᵇ  NSᵃ  NSᵃ  NSᵃ  .86ᵇ  .89ᵇ  .36ᵇ  .36ᵇ  .86ᵇ  .21ᵇ  .54ᵇ |

**P* < .05. ᵃNon-significant, but Chi Square test is not valid due to expected count <5 in several cells, ᵇMann Whitney Uᵇ

**S2 Examination of Anomalous Self-Experience (EASE) domain and items**

*Domain 1 Cognition and stream of consciousness*

Thought interference 1.1

Loss of thought ipseity 1.2

Thought pressure 1.3

Thought block 1.4

Silent thought echo 1.5

Ruminations-obsessions 1.6

Perceptualization of inner speech or thought 1.7

Spatialization of experience 1.8

Ambivalence 1.9

Inability to discriminate modalities of intentionality 1.10

Disturbance of thought initiative/intentionality 1.11

Attentional disturbances 1.12

Disorder of short-term memory 1.13

Disturbance of time experience 1.14

Discontinuous awareness of own action 1.15

Discordance between expression and expressed 1.16

Disturbance of expressive language function 1.17

*Domain 2 Self-awareness and presence*

Diminished sense of basic self 2.1

Distorted first-person perspective 2.2

Psychic depersonalization (self-alienation) 2.3

Diminished presence 2.4

Derealization 2.5

Hyperreflectivity (increased reflectivity) 2.6

I-split (‘‘Ich-Spaltung’’) 2.7

Dissociative depersonalization 2.8

Identity confusion 2.9

Sense of change in relation to chronological age 2.10

Sense of change in relation to gender 2.11

Loss of common sense, perplexity, lack of natural evidence 2.12

Anxiety 2.13

Ontological anxiety 2.14

Diminished transparency of consciousness 2.15

Diminished initiative 2.16

Hypohedonia 2.17

Diminished vitality 2.18

*Domain 3 Bodily experiences*

Morphological change 3.1

Mirror-related phenomena 3.2

Somatic depersonalization (bodily estrangement) 3.3

Psychophysical misfit and psychophysical split 3.4

Bodily disintegration 3.5

Spatialization (objectification) of bodily experiences 3.6

Cenesthetic experiences 3.7

Motor disturbances 3.8

Mimetic experience (resonance between own movement and others’ movements) 3.9

*Domain 4 Demarcation/transitivism*

Confusion with the other 4.1

Confusion with one’s own specular image 4.2

Threatening bodily contact and feelings of fusion with another 4.3

Passivity mood 4.4

Other transitivistic phenomena 4.5

*Domain 5 Existential reorientation*

Primary self-reference phenomena 5.1

Feeling of centrality 5.2

Feeling as if the subject’s experiential field is the only extant reality 5.3

‘‘As if ’’ feelings of extraordinary creative power or extraordinary insight into hidden dimensions of reality 5.4

‘‘As if ’’ feeling that the experienced world is not truly real, as if it was only somehow apparent, illusory or deceptive 5.5

Magical ideas linked to the subject’s way of experiencing 5.6

Existential or intellectual change 5.7

Solipsistic grandiosity 5.8

Definitions of domains, items and subtypes (subtypes not included in S2) are outlined in:

Parnas, J., et al. (2005). "EASE: Examination of Anomalous Self-Experience." Psychopathology **38**(5): 236-258.

**S3 Demographic and clinical characteristics at baseline and follow-up in the CHR and in the non-progressive symptoms group**

| Characteristics | CHR  *N* = 26 | Non-progressive symptoms group  *N* = 6 | *P-*value |
| --- | --- | --- | --- |
| Male, n (%)  Age, mean (SD)  Years of education, mean (SD)  Employed or studying, n (%)  Born in Norway, n (%)  EASE total baseline (lifetime), mean (SD)  EASE total baseline (last year), mean (SD)  EASE total follow-up, mean (SD)  SOPS positive, baseline, mean (SD)  SOPS negative, baseline, mean (SD)  SOPS disorg., baseline, mean (SD)  SOPS general, baseline, mean (SD)  SOPS positive, follow-up , mean (SD)  SOPS negative, follow-up, mean (SD)  SOPS disorg., follow-up, mean (SD)  SOPS general, follow-up, mean (SD)  GAF-F, baseline, mean (SD)  GAF-F, follow-up, mean (SD)  Transition to psychosis, n (%)  Full remission, n (%)  SPD baseline, n (%)  SPD follow-up, n (%) | 15 (58.0)  19.0 (3.4)  11.4 (1.8)  15 (58.0)  23 (88.5)  15.19 (8.81)  13.77 (8.73)  11.27 (10.40)  10.77 (3.63)  12.38 (6.95)  6.81 (3.49)  7.81 (3.11)  6.38 (5.94)  9.38 (6.81)  4.96 (4.53)  5.12 (3.54)  56.42 (10.63)  60.46 (15.55)  4 (15.4)  9 (34.6)  4 (15.4)  6 (23.1) | 6 (100)  24.2 (2.8)  12.8 (1.2)  2 (33.3)  6 (100)  15.83 (4.54)  13.83 (4.62)  10.33 (9.03)  8.83 (2.04)  13.00 (7.95)  7.33 (3.01)  6.67 (4.27)  7.33 (3.93)  12.33 (9.81)  5.83 (3.54)  4.33 (3.50)  55.83 (12.70)  56.67 (17.87)  0  2 (33.3)  1 (16.7)  3 (50.0) | *NS*ᵃ  .002ᵇ*  .03ᵇ*  *NS*ᵃ  *NS*ᵃ  .72ᵇ  .83ᵇ  .94ᵇ  .16ᵇ  .91ᵇ  .69ᵇ  .52ᵇ  .44ᵇ  .44ᵇ  .49ᵇ  .72ᵇ  .76ᵇ  .62ᵇ  *NS*ᵃ  *NS*ᵃ  *NS*ᵃ |

**P* < .05. ᵃNon-significant, but Chi Square test is not valid due to expected count <5 in several cells, ᵇMann Whitney U Test ᵇ
